# Smart logistics of perishable goods: emerging nanomaterial-based sensing technologies for real-time monitoring and analytical control

**DOI:** 10.1186/s12951-026-04340-2

**Published:** 2026-04-04

**Authors:** Haibin Liu, Gao Zhou, Zhili Wang, Xueju Li

**Affiliations:** 1 Institut Montpellier Management, University of Montpellier, Montpellier, 34960 France; 2 School of Management, Hunan University of Information Technology, Changsha, 410151 Hunan Province China; 3 Graduate School of International Studies, Yonsei University, Seoul, 03722 South Korea; 4 School of Economics and Management, Daqing Normal University, Daqing City, 163712 Heilongjiang Province China; 5 Department of Information Engineering, Guizhou Light Industry Polytechnic University, Guiyang, 550003 Guizhou Province China

**Keywords:** Intelligent packaging, Internet of Things, Cold chain, Volatile organic compounds, Shelf life prediction

## Abstract

Perishable supply chains lose substantial value because product deterioration is rarely measured directly during distribution, while static date labels and temperature records provide only indirect assurance of quality. This review surveys nano-enabled sensor elements that translate headspace chemistry and microbial activity into actionable freshness information, prioritizing studies evaluated on real foods and under realistic cold chain excursions. We synthesize progress across three technology clusters. Optical indicators combine pH responsive dyes, polydiacetylene assemblies, quantum dots, and carbon dots to deliver visible or camera-readable outputs that track amine accumulation and ripening-related metabolites. Gas sensors and electronic noses employ semiconducting metal oxides, graphene, and carbon nanotube networks to quantify volatile organic compounds (VOCs) such as ammonia, trimethylamine, ethylene, and carbon dioxide, with increasing attention to room temperature operation and pattern-based classification. Biosensors extend specificity through antibodies, aptamers, and molecularly imprinted polymers on conductive nanostructures, frequently using electrochemical impedance spectroscopy (EIS) to detect bacteria, toxins, or enzymatic byproducts at low loads. Integration pathways are reviewed from passive color labels to wireless tags using radio frequency identification (RFID), near field communication (NFC), and Bluetooth Low Energy (BLE), enabling package-level data capture and cloud analytics for remaining shelf-life estimation. We also consolidate cross-cutting constraints including calibration drift, humidity cross sensitivity, limited independent validation, added system cost, and safety and regulatory requirements around nanomaterial migration. Taken together, the literature indicates that multi-parameter designs coupled to data models are central to translating laboratory sensitivity into deployable decision support in practice.

**Graphical Abstract:**

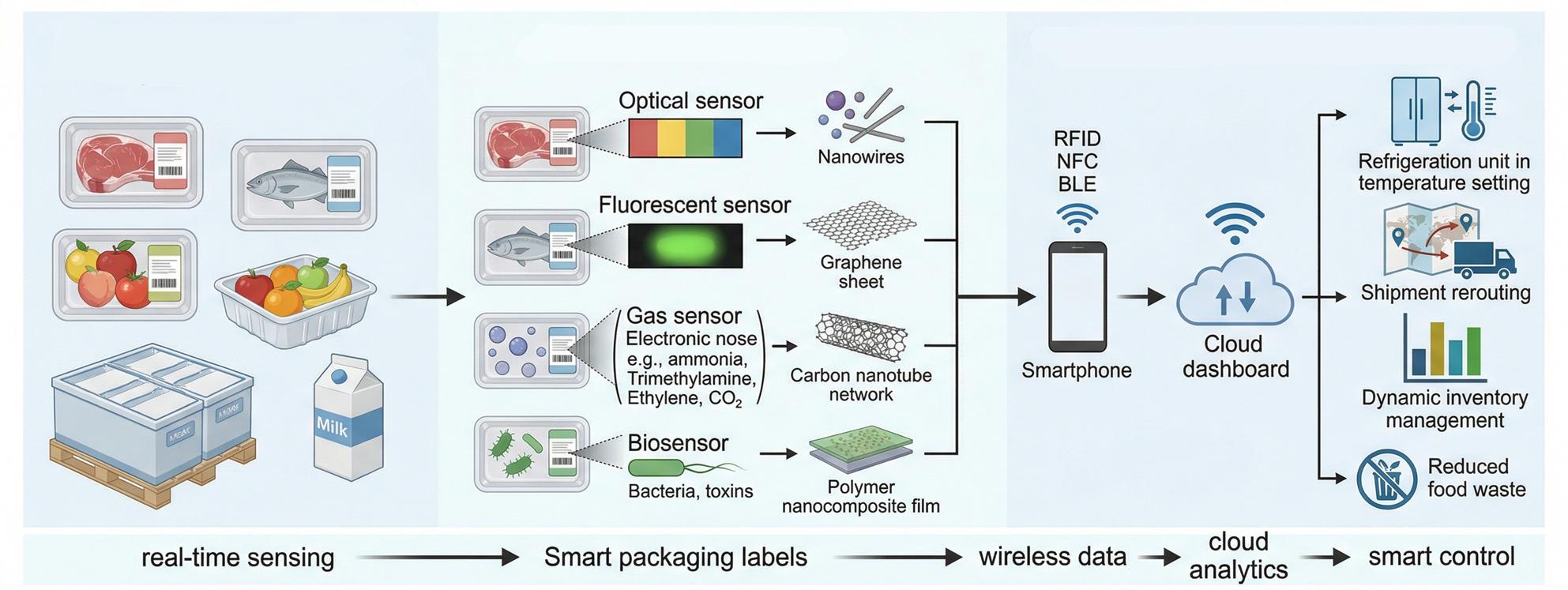

## Background

Ensuring the quality and safety of perishable goods through the supply chain is a global challenge. Roughly one-third of all food produced is lost or wasted before consumption [1]. Much of this waste is due to spoilage that goes undetected until it’s too late, or overly conservative expiration dates that prompt disposal of still-edible products [2, 3]. Traditional cold-chain logistics rely on fixed “best-by” dates and rudimentary monitoring like temperature loggers. However, static date codes cannot account for real-time variations in storage conditions, and even a well-maintained cold chain cannot reveal subtle deterioration in a product’s internal quality [4, 5]. As a result, distributors and retailers often discard shipments at the first hint of potential spoilage, erring on the side of caution and contributing to food waste. This reactive approach costs the industry billions and undermines food security [6].

Smart logistics systems seek to tackle these problems by combining real-time sensing with data-driven decision-making. The emergence of nanomaterial-based sensors offers a promising path to monitor perishable goods continuously from farm to fork. Nanomaterials, including metal nanoparticles, metal oxides, carbon nanostructures, and polymer nanocomposites, exhibit distinctive optical, electrical, and catalytic properties that emerge at the nanoscale. When integrated into biosensors and physicochemical sensors, these nanomaterials enable detection of minute changes in the product’s environment or composition, acting as electronic “noses” and “tongues” to sniff out spoilage indicators well before human senses can [7]. Unlike conventional lab tests which are slow and require sampling, nanosensors can be embedded directly in packaging or storage containers to provide on-line measurements of freshness markers such as gases, metabolites, pH changes, or microbial metabolites [8]. Recent advances have produced smart labels and tags incorporating nano-engineered sensing elements with wireless readout (e.g. RFID/NFC tags or smartphone-scannable sensors) that can accompany each package through the supply chain [9]. These developments transform passive packaging into an active monitoring system, laying the foundation for dynamic shelf-life management and “intelligent” distribution decisions.

**Fig. 1:**
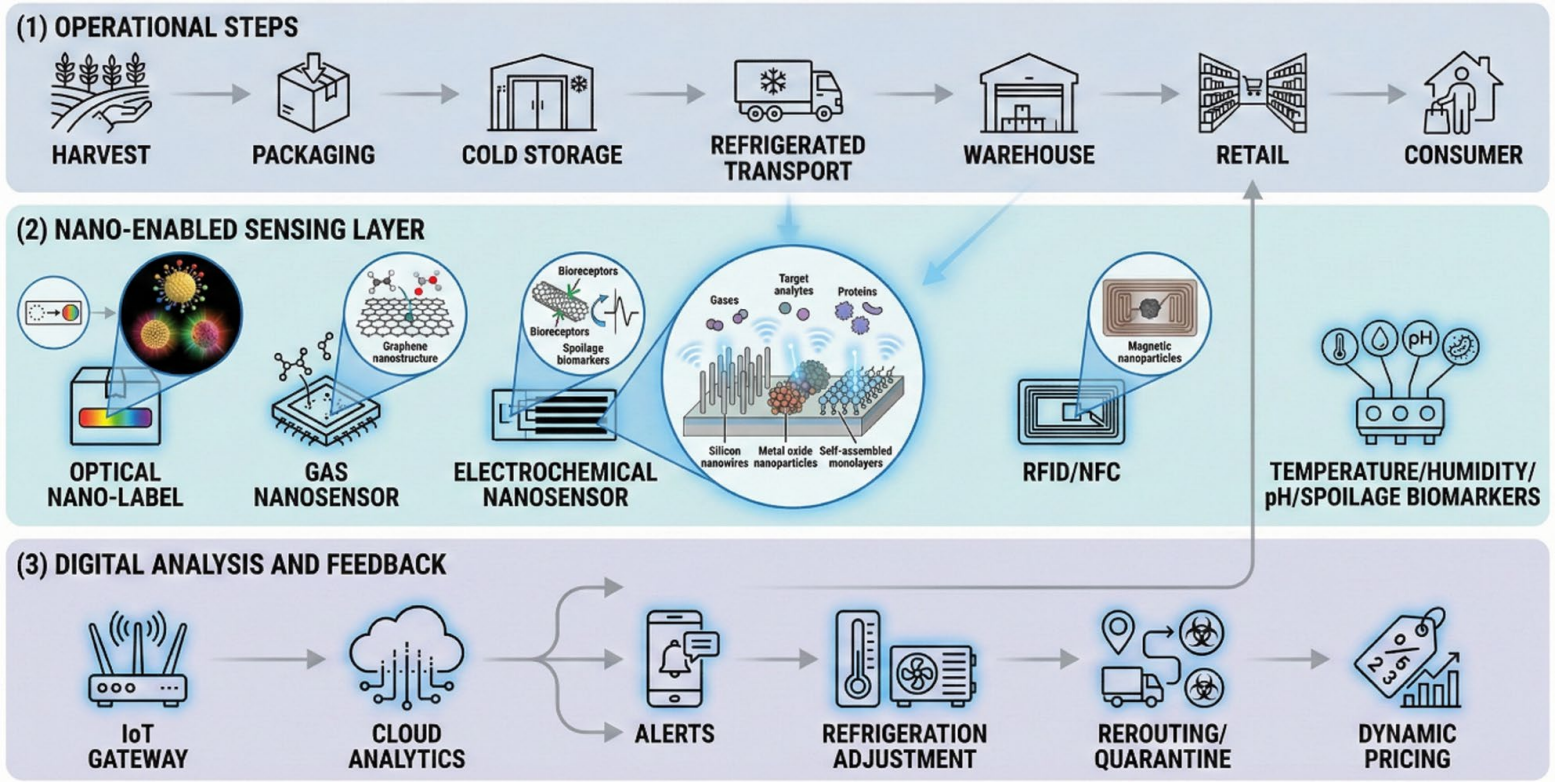
Layered framework of nano-enabled monitoring, analytics, and feedback control in smart cold-chain logistics for perishable goods

Crucially, nanotechnology-based sensors can do more than merely detect spoilage. They enable analytical control by generating real-time data that stakeholders can use to make timely, informed interventions. For example, continuous monitoring of temperature, humidity, and biochemical spoilage signals allows logistic managers to adjust refrigeration or reroute shipments if thresholds are crossed, thus preventing quality loss [10]. Likewise, retailers equipped with real-time freshness data can implement dynamic pricing or stock rotation to ensure older inventories are sold or processed first, reducing waste [11]. In essence, these sensors create a feedback loop: the conditions and quality of the product guide interventions in storage and handling, rather than blindly following pre-set schedules. The integration of nanosensors with Internet-of-Things (IoT) platforms and cloud analytics further amplifies this potential by enabling supply chain-wide visibility and automated decision algorithms. Figure 1 summarizes the end-to-end concept of nano-enabled smart logistics for perishables, linking in situ sensing at multiple supply-chain nodes with wireless readout, IoT and cloud analytics, and closed-loop interventions for analytical control. This framework highlights how real-time freshness signals can be translated into actionable decisions such as refrigeration adjustment, shipment rerouting, and dynamic inventory management.”

This review provides a comprehensive and critical overview of “Smart logistics of perishable goods: emerging nanomaterial-based sensing technologies for real-time monitoring and analytical control.” We first examine the unique challenges in perishable goods logistics and the limitations of current monitoring methods, to underscore the need for nano-enabled smart sensors. We then categorize the major nanomaterial-based sensor technologies under development for real-time quality monitoring, including optical sensors (colorimetric and fluorescent), gas sensors, and biosensors, and explain how nanomaterials provide enhanced sensitivity and added functionality. Specific examples from recent literature are analyzed to illustrate sensor performance and practicality. Next, we discuss strategies for integration into smart packaging and IoT networks, detailing how sensor data is collected and utilized in a logistics context. In the penultimate section, we turn to a critical discussion of the pros and cons of these emerging technologies. We dissect core challenges, such as validation of sensor accuracy, cost and scalability, regulatory and safety concerns, and stakeholder acceptance, alongside the arguments of proponents who see nano-sensors as a revolution for reducing waste and enhancing safety. Key points of controversy and debate in the field are identified. Finally, we outline future prospects and research directions needed to address current shortcomings and fully realize the potential of nanotechnology in smart logistics of perishables. By surveying over 100 recent studies and industry reports, this review aims to provide both depth and breadth: a state-of-the-art summary of technological advances and a balanced critique of their feasibility and impact. The insights herein are particularly relevant to food scientists, packaging engineers, supply chain managers, and regulators interested in harnessing innovative sensing for a more sustainable and safe food system.

Table 1 summarizes the main classes of nanomaterials being employed in food-sensing applications, their typical sensing mechanisms, and example targets. This provides context for the sensor technologies detailed in later sections. This table highlights how diverse nanomaterials contribute to various sensing approaches. Many sensor designs actually integrate multiple nanomaterials to achieve synergistic effects. For instance, a hybrid sensor may use magnetic nanoparticles coated with gold, combining magnetic capture of bacteria with plasmonic optical readout. Another example is a graphene sheet decorated with silver nanoparticles, leveraging graphene’s electrical conductivity and Ag’s antimicrobial and colorimetric properties in one platform. The choice of nanomaterial is guided by the target analyte and food matrix: in situ sensors that contact food often favor biocompatible polymers or oxide particles, whereas external sensors or test strips can exploit a broader range of inorganic nanomaterials for stronger signals. As we delve into each sensing modality below, we will see how these nanomaterials are engineered into devices that fit seamlessly into the logistics of perishable products, enabling a new era of smart, responsive supply chains.

**Table 1:** Overview of major nanomaterial classes used in food sensory systems, their sensing mechanisms, and representative applications in perishable goods monitoring

Nanomaterial class	Main sensing mechanisms	Representative targets/applications	References
Metal nanoparticles (Au, Ag)	Plasmonic colorimetric shifts (localized surface plasmon resonance); catalytic nanozyme activity (peroxidase-like) enabling color changes; redox-active surfaces for electrochemical signals	Chemical adulterants & toxins: e.g. gold NP aggregation assays for melamine in milk (color change indicating ppm-level melamine) [12]. Peroxide/oxidation indicators: nanozyme-based colorimetric tests for food spoilage (H_2_O_2_ from oxidation) [9]	[9, 12]
Magnetic nanoparticles (Fe_3_O_4_, ferrites)	Immuno-magnetic capture of pathogens (magnetic NPs coated with antibodies concentrate bacteria on sensor) boosting detection; magneto-electrochemical readouts (e.g. frequency shifts in piezo sensors); catalytic degradation of target molecules	Pathogen detection: magnetic-NP immunosensors for rapid E. coli or Salmonella detection on food surfaces. Contaminant cleanup and sensing: magnetic NPs pulling pesticide residues or toxins from samples for analysis	[9]
Metal oxides & ceramics (ZnO, TiO_2_ SnO_2_ SiO_2_)	Chemiresistive gas sensing – surface adsorption of gases changes electrical resistance; photo-activated sensing (UV illumination changes conductivity); inherent antimicrobial activity in packaging films	Volatile spoilage gases: e.g. ZnO nanowire sensors for ammonia (fish/meat spoilage) and H_2_S [13]; SnO_2_-based sensors for ethylene (fruit ripening). Active packaging: TiO_2_ in film releasing antimicrobial ROS under light to delay spoilage [13]	[14, 15]
Carbon nanomaterials (graphene, CNTs, carbon dots)	High-surface-area conductive networks for electrochemical sensors (voltammetry, impedance); chemoresistive detection of gases/vapors; fluorescence quenching and emission (quantum confinement in carbon dots)	Electrochemical freshness sensors: graphene-modified electrodes detecting spoilage acids or sugars (current change). Electronic noses: carbon nanotube sensor arrays distinguishing meat freshness by VOC pattern. Fluorescent tags: carbon dot sensors that glow or dim in presence of specific amines, readable by smartphone camera	[9]
Quantum dots (QDs) (ZnS, CdSe, carbon QDs)	Fluorescence emission in visible range, tunable by QD size; quenching or intensity changes when QD interacts with target (e.g. sulfur compounds quench fluorescence). Ratiometric fluorescence possible for self-calibrating sensors	Biogenic amine sensors: e.g. a dual-emission QD probe that changes fluorescence ratio in presence of trimethylamine from fish spoilage. Metal contaminant sensing: QDs functionalized to detect heavy metals or toxins in food (fluorescence turn-off)	[14, 16]
Polymer nanomaterials (polydiacetylene, molecularly imprinted polymers – MIPs, dendrimers)	Colorimetric transformations: e.g. polydiacetylene (PDA) polymers that visibly turn from blue to red upon binding amines or when pH rises. Synthetic receptors: MIPs engineered with nanocavities that bind a target and produce an electrochemical or optical change	Freshness indicators: PDA-based coating or beads in meat packaging that change from blue to red as total amine levels increase with spoilage. Chemical contaminant detection: MIP-functionalized electrodes that selectively bind a food adulterant (e.g. antibiotic or pesticide) and yield a measurable impedance change	[17]

## Perishable logistics and the need for real-time monitoring

Perishable goods logistics is constrained by time and environmental conditions. Quality degradation begins immediately after harvest or production: microbial growth, enzymatic activity, and chemical breakdown lead to loss of freshness, off-odors/flavors, and potentially hazardous spoilage byproducts. Traditionally, the cold chain is employed to slow these processes. However, proper temperature control alone is not a complete safeguard. Foods may still spoil even when stored within recommended ranges due to factors such as high initial microbial load or inherent perishability. Conversely, some foods can remain safe and of acceptable quality slightly beyond their printed expiration date when stored under optimal conditions [18].

Conventional monitoring in supply chains is largely limited to tracking external conditions (temperature, humidity) rather than the internal quality of the product. Data loggers and thermometers placed in storage areas or transport containers provide coarse assurance that, say, milk was kept below 4 °C. But they cannot detect a localized temperature abuse, nor can they reveal if microbial spoilage is already at an advanced stage due to pre-existing contamination [19]. Furthermore, logistics operators and retailers rely on conservative shelf-life models that assume worst-case handling. As Weis (2021) notes, fixed date labels do not account for deviations in handling. When a product is exposed to non-recommended conditions, there may be “no way to know it,” and the date label can lose practical meaning [20]. The result is a system that often errs on the side of caution by discarding food early [21, 22], contributing to staggering waste. For example, store surveys have found thousands of dollars’ worth of meat thrown away weekly per supermarket due to expiry and appearance concerns [23, 24]. Clearly, more dynamic and product-specific freshness indicators are needed to support better decision-making.

Smart monitoring addresses this need by measuring indicators of spoilage or quality directly from the product or its atmosphere in real time. Key spoilage indicators include: temperature abuse history, pH changes (from microbial metabolites or chemical reactions), and most prominently volatile organic compounds (VOCs) produced by spoilage organisms. In meats and fish, bacteria generate volatile amines (ammonia, trimethylamine, cadaverine, putrescine, etc.) as proteins decompose, which cause the characteristic “off” odors of spoilage [16, 25, 26]. In fruits, ripening and overripening are associated with ethylene gas emission and increased CO₂, as well as fermentation alcohols if decay sets in. Moisture and gas composition in packaging also shift: for instance, O₂ may drop and CO₂ rise in a sealed produce bag due to respiration. All these are measurable phenomena. Traditional quality tests would require taking a sample to a lab, which is impractical in a fast-moving supply chain. This is where in-package sensors become valuable. By continuously monitoring headspace gases or the product itself, they effectively convert each package into a self-monitoring unit.

Importantly, real-time monitoring enables analytical control by allowing timely intervention. For instance, if a sensor in a case of fish detects an abnormal rise in ammonia that signals accelerated spoilage, the batch can be redirected for immediate sale or processed into cooked products instead of being stored for a later date [27, 28]. Data analytics can predict remaining shelf life: BlakBear’s system correlates sensor readings with bacterial growth models to forecast how many days of freshness remain [29, 30]. This enables dynamic expiration dating, so each package can follow its own product-specific use-by timeline rather than relying on a single fixed date. The benefits are twofold: it can reduce waste by preventing premature disposal of still-safe products, while also strengthening safety assurance by flagging genuinely spoiled items even when the printed date has not yet passed. Field trials underscore these benefits: For instance, intelligent packaging with pH-responsive indicators has been shown to reduce premature disposal by ~ 20% in test settings by giving more accurate freshness info than static labels [31]. Another estimate suggests supply chain-wide freshness monitoring could extend shelf life by at least one day for poultry, significantly cutting store waste [32].

From an operations perspective, implementing real-time monitoring in logistics means equipping shipments with sensors and establishing data communication infrastructure. The advent of low-cost flexible electronics and wireless technologies (RFID, BLE, etc.) makes it feasible to gather sensor data at scale. Modern warehouses and vehicles can be fitted with RFID readers that automatically scan sensor-integrated packages moving through, uploading their readings to cloud databases [33, 34]. There are already IoT platforms where a manager can view a “freshness dashboard” of all products in storage, much like one would track temperature or inventory counts [35, 36]. Such visibility was unthinkable a decade ago. In effect, each perishable item can have a digital twin representing its quality status. This aligns with broader trends in supply chain digitalization and traceability [37, 38]. A major driver for these efforts is not only reducing waste, but also strengthening food safety and compliance. Being able to demonstrate that products were maintained under appropriate conditions and withdrawn promptly when spoilage occurs is valuable for regulatory requirements and for protecting the brand in the event of recalls.

Despite clear advantages, the transition to sensor-driven logistics is still in early stages. Historically, simpler devices like Time-Temperature Indicators (TTIs) have been available. For example, stickers that irreversibly change color when cumulative thermal exposure exceeds a threshold, thereby indicating potential shelf-life exhaustion. While useful, TTIs only reflect temperature history, not whether the food actually spoiled and can be prone to false positives/negatives if the product’s actual spoilage kinetics differ [45]. By contrast, nanomaterial sensors that detect actual spoilage metabolites or microbes provide a more direct measurement of food condition. Over the past few years, several proof-of-concept smart packaging systems have emerged. For example, Mimica Touch is a bio-inspired label that becomes bumpy as food spoils, using a gel that changes its physical state over time. Although it can be calibrated to match the shelf life of products such as milk, it does not directly measure microbial growth and instead approximates cumulative time–temperature exposure. By contrast, newer generations of nanosensors target specific chemical signatures associated with freshness and spoilage. For instance, Professor Güder’s group at Imperial College developed a paper-based RFID sensor that directly detects ammonia and amine vapors from meat spoilage, and transmits an electrical signal that can be read by a smartphone [39]. This sensor is highly sensitive, detecting ammonia down to approximately 0.2 ppm, which is far below the human nose’s roughly 20 ppm detection threshold [40]. It also responds within minutes to concentration changes, effectively serving as a digital nose within each package.

**Fig. 2:**
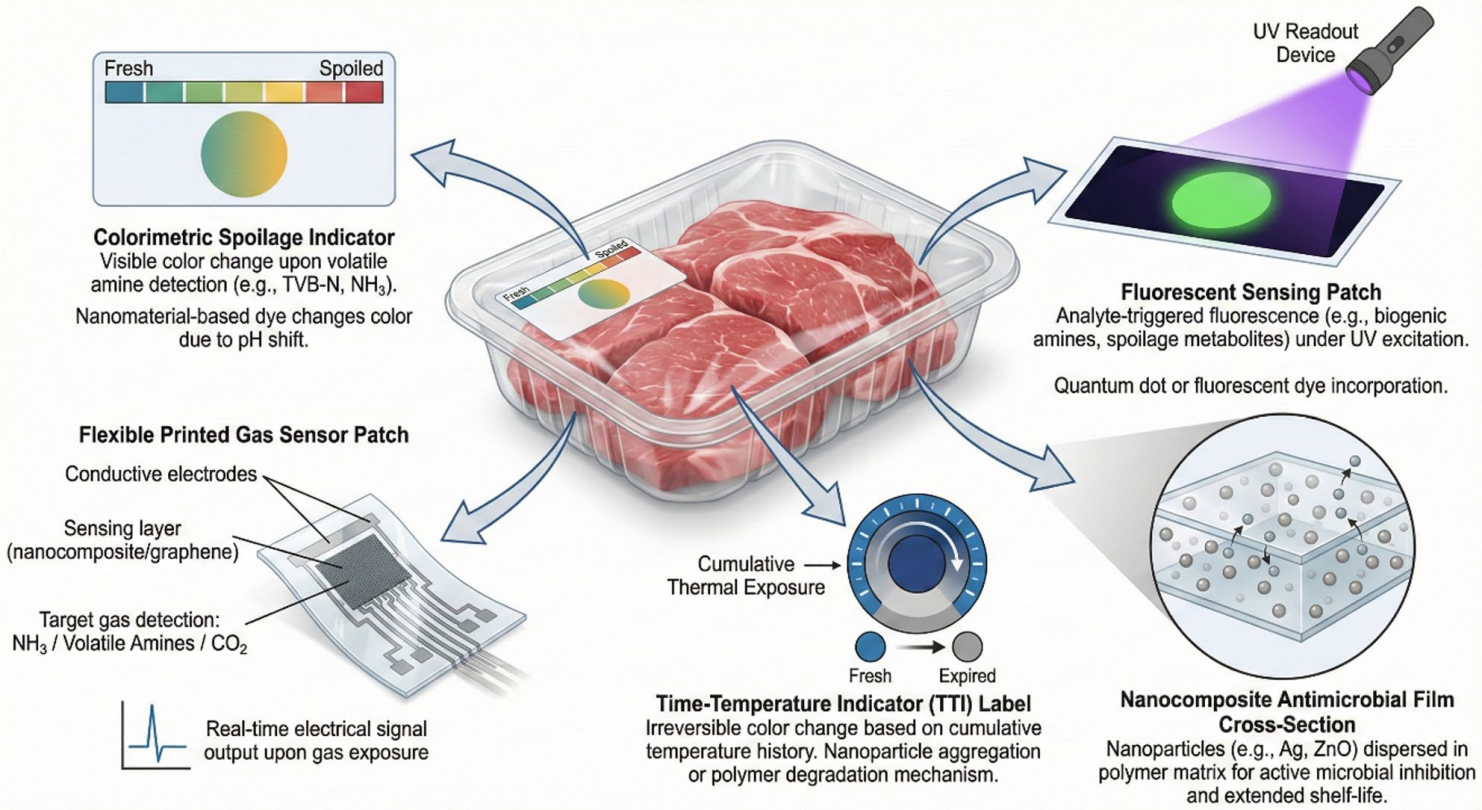
Schematic of a nano-enabled smart packaging architecture for fresh meat, integrating direct spoilage sensing (colorimetric, fluorescent, and gas-responsive labels), proxy monitoring of cumulative thermal exposure (TTI), and nanocomposite active film functionality

As illustrated in Fig. 2, nano-enabled smart packaging is presented here as an integrated, package-level sensing architecture for fresh meat, in which multiple functional components are attached to or embedded within the package in the form of labels, patches, and nanocomposite films. In this configuration, direct chemical readouts of spoilage (e.g., pH-responsive color change, volatile amine or CO₂ sensing, and fluorescence-based metabolite detection) are combined with proxy indicators of risk accumulation, such as time–temperature exposure, and with active material functions that can retard deterioration. This layout emphasizes the operational value of multi-parameter sensing in perishable logistics, because no single indicator is sufficient under all storage and distribution conditions. For example, time–temperature indicators can effectively track cumulative thermal abuse, but they do not directly verify biochemical spoilage, whereas gas- or metabolite-responsive labels provide more direct evidence of product degradation but may be influenced by package atmosphere, headspace volume, and food matrix effects. By integrating complementary sensing modalities within realistic packaging materials, the system can support more robust estimation of remaining shelf life and reduce false-positive or false-negative decisions in downstream handling. Importantly, the figure also highlights a key design principle of nano-enabled packaging: nanomaterials are engineered not only to improve sensitivity, but also to provide selectivity, rapid response, and compatibility with scalable low-power readout formats, including printed electronics and wireless identification platforms.

## Nanomaterial-based sensor technologies for perishable goods

Emerging sensors for perishable goods leverage nanomaterials to detect a range of spoilage indicators with high sensitivity. These sensors can be broadly categorized by their detection mode: optical sensors, electrochemical sensors, and biorecognition sensors [41–43]. Often, a single device may involve multiple transduction modes. Below, we examine key examples in each category, illustrating how nanomaterials enable the sensing mechanism. Particular attention is given to sensors that have been tested on actual food items or in simulated supply-chain conditions, as these offer the most insight into practical performance. Mechanistically, ‘nano-enablement’ most often increases signal-to-noise by (i) amplifying analyte–surface interactions via high specific surface area and nanoscale porosity, (ii) converting weak chemical events into strong electrical/optical changes through charge transfer and band-bending (e.g., depletion-layer modulation in semiconducting metal oxides), (iii) accelerating interfacial reaction kinetics through catalytic/electronic sensitization (e.g., noble-metal decoration), and/or (iv) enhancing transduction efficiency by creating conductive percolation networks (CNT/graphene) in which small adsorption- or swelling-induced perturbations produce large resistance shifts. In optical labels, nanocarriers and nanocomposite matrices additionally improve practical sensitivity by reducing dye leaching, controlling local microenvironment (pH/ionic strength), and sharpening transitions through confinement and mass-transport engineering.

### Optical sensors: colorimetric and fluorescent indicators

Optical freshness indicators are among the most user-friendly smart packaging elements because they provide an intuitive visual signal of spoilage that can be interpreted without any special equipment. Colorimetric sensors, in particular, have seen extensive research. The principle is simple: a dye or pigment that changes color in response to a chemical trigger is embedded in the package. Nanomaterials come into play by either carrying these dyes or enhancing their response. At the mechanistic level, nano-incorporation can (i) increase effective analyte uptake by providing high-area adsorption sites for volatile bases (e.g., NH₃/TMA) and moisture, (ii) localize and stabilize the chromophore in a confined microenvironment that suppresses diffusion/leaching, and (iii) sharpen the apparent transition by coupling the dye to pH-active or Lewis-acid/Lewis-base nanosurfaces that accelerate local proton-transfer equilibria. In practice, these effects improve the slope of the color-change response versus amine exposure and reduce baseline drift during storage, which is critical for logistics timelines. A prominent example is the use of anthocyanins integrated into a biopolymer film. Anthocyanins are pH-sensitive pigments that shift from purple-red under acidic conditions to greenish-yellow under more basic conditions. As meat and fish spoil, alkaline amines accumulate and raise the local pH, so anthocyanin-based films can gradually change color as spoilage progresses [44, 45]. To improve this concept, researchers have loaded anthocyanins onto ZnO nanoparticles or into nanofiber matrices, which both stabilizes the pigment and amplifies the color change. ZnO itself is a nanomaterial that can react with acids/bases and influence pH; one study showed that incorporating ZnO nanorods in an anthocyanin film made the color transition sharper and allowed detection of fish spoilage a day earlier than a film without ZnO [17, 46]. The high surface area of nanomaterials means more interaction with volatile amines, thus a more pronounced color shift for a given amine concentration.

One particularly striking colorimetric approach uses polydiacetylene (PDA), a conjugated polymer that can be engineered to switch from blue to red in response to specific molecular interactions or environmental changes such as temperature or pH. PDA-based sensors are often formulated as hydrogel beads or thin films. Jang et al. (2022) developed PDA-containing hydrogel beads that remain blue when fresh but turn reddish-pink as meat spoils and releases amines, consistent with amine-induced perturbation of PDA’s conjugated backbone (via headgroup interactions and local packing stress) that drives the blue-to-red chromatic transition [17]. These beads can simply be placed in a package or even mixed into a packaging film. During tests with pork, the color change correlated well with bacterial growth over 4 days at room temperature, providing a real-time visual freshness indicator [47]. Because the PDA transition is irreversible and highly visible, it’s ideal for use-by warnings. Nanomaterials contribute here insofar as PDA itself is often prepared in nano-assemblies (e.g., liposomes) to maximize surface exposure. Additionally, combining PDA with silver nanoparticles has been shown to tune its sensitivity. Silver nanoparticles can promote the polymer’s color transition by facilitating reactions and interactions with amines [48, 49]. Such hybrid sensors (PDA + AgNP) have been reported in time–temperature indicators as well, where thermal exposure triggers a color shift that is expedited by the presence of plasmonic nanoparticles [50].

Colorimetric indicator labels are attractive because of their simplicity, since no dedicated reader is strictly required. For quantitative tracking, however, images can be captured and analyzed with software or a smartphone application to translate color intensity into numerical information. They are already being piloted in commercial settings. For instance, Insignia Technologies markets an OnVu label and other firms are testing anthocyanin labels for meat packaging. However, these early products often lack the precision to be trustworthy alone; this is where nanotech enhancements aim to make the difference by improving response specificity and sensitivity. Academic research consistently shows that nano-enabled films have clearer color changes at earlier stages of spoilage compared to non-nano versions [51].

In addition to static color changes, some optical sensors use fluorescence, which can be even more sensitive but normally requires an excitation source (like a UV lamp or LED) and a detector. Quantum dot (QD) nanomaterials and carbon dots are prominent in this area. These are nanoscale semiconductors that emit bright fluorescence of tunable color. In a fresh state, the fluorescence may be strong, but if they interact with a target compound, the fluorescence might quench (dim) or shift wavelength. For example, Sun et al. (2025) developed a dual-mode sensor using a fluorescent dye that emits red fluorescence when protonated, and loses fluorescence when exposed to biogenic amines [16]. They embedded this dye in a label and used a smartphone camera under a UV lamp to quantify fish freshness (salmon) by analyzing the red/blue color channels of the label’s image [52]. The nanomaterial aspect in that case was the dye’s crystalline nanostructure which provided a fast 5-second response and a low detection limit (~ 5 µM trimethylamine) [53]. Another example is the use of upconversion nanoparticles functionalized to detect spoilage. These have been researched to create sensors that could be read by simple IR readers, removing the need for UV.

Optical sensors, whether colorimetric or fluorescent, benefit greatly from nanomaterials in terms of stability and tunability. Many natural dyes can leach or degrade, but encapsulating them in nanoparticles or tethering them onto nanosurfaces can mitigate this [14, 54]. Furthermore, by mixing different dyes or QDs, one can create a sensor that gives a distinct optical signature for different levels of spoilage, sometimes called optical barcode sensors. For instance, an array of colorimetric spots can together act as an “electronic tongue” or “electronic nose,” with a pattern of color changes that can be analyzed to determine overall spoilage levels [55, 56]. Suslick and colleagues have pursued this approach using nanoporous pigments that change color when exposed to various gas mixtures, creating a sort of color fingerprint of the food’s aroma [57]. While highly informative, these multi-dye sensor arrays may be more suited to backend quality control than as consumer-facing labels, due to their complexity in interpretation.

In summary, optical nanosensors provide an accessible route for freshness monitoring because the readout can range from naked-eye color change to simple smartphone-assisted image analysis. Their strongest practical advantage is low operational complexity, particularly for colorimetric labels that do not require sample pretreatment and can often function as package-integrated indicators. However, this apparent simplicity depends strongly on material design: bio-based matrices such as alginate, anthocyanin-containing films, and other biodegradable polymers improve sustainability and may reduce consumer concern, but they can also be more sensitive to humidity, dye migration, and storage instability than fully synthetic supports. Fluorescent formats generally improve sensitivity and permit quantitative analysis, yet they also increase instrument dependence because excitation sources and optical capture are required. Accordingly, the main translational challenge for optical sensors is not only sensitivity, but establishing a robust and reproducible correlation between color/fluorescence response, validated spoilage indices, and actionable control thresholds for sale, rerouting, or disposal.

### Gas sensors and electronic noses

Many perishables emit a complex mixture of gases as they age. Gas sensors that detect this spoilage volatiles are central to smart logistics, essentially acting as automated noses. Nanomaterials have revolutionized gas sensing by providing high surface area, tunable surface chemistry, and novel conduction mechanisms that make sensors more sensitive and selective to trace gases. The most widely studied are chemiresistive sensors, typically using semiconducting metal oxide nanostructures. Materials like SnO₂, ZnO, or WO₃ in nanoparticle or nanowire form can adsorb gas molecules on their surfaces; this adsorption leads to electron transfer or scattering that changes the material’s electrical resistance. More specifically, in air, adsorbed oxygen species extract electrons from n-type SMOx and form an electron-depletion layer (band bending). Exposure to reducing spoilage volatiles (e.g., NH₃/TMA) consumes/reacts with these surface oxygen species and/or donates electrons, decreasing depletion width and intergrain barriers, which produces a measurable drop in resistance. Nanoscaling increases the fraction of the material within the space-charge region, so a given surface reaction yields a larger relative conductance change (i.e., higher sensitivity). By measuring the resistance change, one can infer the gas concentration [58, 59]. For example, when ZnO nanowires adsorb ammonia (NH₃), electron donation from NH₃ increases the carrier concentration in ZnO and lowers its resistance. The extent of this resistance decrease correlates with the NH₃ concentration. Spoiling fish or meat can generate NH₃ in the low ppm range, which ZnO or SnO₂ nanosensors can detect well below [17, 60, 61]. Metal oxide sensors are relatively mature, but nanostructuring them vastly improves their detection limits because nanostructures have a much larger fraction of atoms on the surface ready to interact with gas molecules.

To illustrate, Yavuzer et al. [17] built a low-cost electronic nose for fish quality monitoring using a set of metal oxide sensors and could correlate the sensor readings with fish freshness indices. Their approach showed that even inexpensive MOS (metal-oxide-semiconductor) sensors, when used in an array, could determine if fish was fresh, semi-fresh, or spoiled with decent accuracy, offering a potential tool for quality checks in warehouses. However, standalone metal oxide sensors often face selectivity challenges. For example, SnO₂ can respond to a range of reducing gases such as ethanol or carbon monoxide, rather than being specific to fish-derived amines. To enhance selectivity, researchers incorporate catalytic nanoparticles onto the oxide surface which preferentially catalyze certain gas interactions [62]. Also, operating temperature is important: many oxide sensors need to run at 200–300 °C to achieve stable baseline and responsiveness, which is not ideal inside food packages. Recent work with nano-oxides and alternative materials aims for lower operating temperatures or even room-temperature sensing by using novel nanostructures that have inherently higher conductance change upon gas binding [63].

Beyond metal oxides, carbon nanomaterials are playing a big role in gas sensing for smart packaging. Carbon nanotubes (CNTs) and graphene are highly responsive to charge-transfer interactions with gas molecules. When species such as NH₃ or NO₂ adsorb onto a graphene surface, they can induce measurable changes in conductivity. Unlike metal oxides, many carbon nanomaterial sensors can operate at room temperature, which is a huge advantage for packaging. Printed networks of single-walled CNTs have been used as general spoilage sensors; by functionalizing the CNT surface with certain groups, they can be made more sensitive to amines. One commercial entity, C2Sense, has developed printable CNT-based gas sensors for ethylene (to monitor fruit ripening) and amines (for meat freshness), using a combination of polymers and nanotubes. In polymer–CNT composite chemiresistors used for amines/ethylene, the polymer acts as a preconcentrator and selectivity layer, while the electrical signal arises from changes in CNT junction barriers and percolation pathways driven by (i) analyte-induced charge transfer and (ii) polymer swelling that modulates intertube spacing; both mechanisms can yield large resistance shifts at room temperature, which is advantageous for smart packaging. Graphene field-effect transistors (FETs) provide another elegant design. A single graphene layer can serve as the transistor channel, and adsorption of target molecules alters the charge carrier density, thereby shifting the device’s electrical characteristics. Researchers have shown graphene FET sensors that can detect 1 ppm of trimethylamine within seconds, and these can be integrated on small RFID-like circuits. For instance, one prototype involved a little graphene sensor connected to an NFC (near-field communication) chip; when placed in a meat container, one could simply tap the smartphone to the package and the sensor’s reading would be transmitted to the phone [17]. This exemplifies the synergy of nanotech sensors with wireless tech to create “smart tags.”

In laboratory comparisons, nanosensors often greatly outperform the human nose and conventional methods in detecting early spoilage. As mentioned, a paper-nanocomposite sensor can detect ~ 0.2 ppm ammonia, whereas humans typically notice fishy odor around 10–20 ppm [64]. This means the sensor can raise an alert before a human would consider the product spoiled, potentially allowing intervention to use the product while it’s still acceptable. However, as Suslick and colleagues caution, sensors should not be “too sensitive” in the sense of triggering alarms for benign fluctuations. At minimum, their outputs should be interpreted in context, for example by distinguishing minor quality loss from conditions that indicate genuine safety risk [65]. For this reason, arrays of multiple sensor types can provide richer data to better assess the spoilage stage, rather than a single gas threshold. Some electronic-nose devices integrate not only chemical sensors but also complementary modalities, such as miniature MOS acoustic sensors to capture popping sounds associated with gas release and humidity sensors to track evaporation. This multi-sensor integration helps build a more complete, context-aware picture of the food environment.

Nanomaterials are even enabling selective gas sensing, which has been a challenge. By molecularly imprinting polymers on CNTs or decorating graphene with specific receptors, researchers aim to differentiate, say, putrescine vs. cadaverine in a complex mixture. Although achieving complete specificity is challenging because gases diffuse freely and complex mixtures can influence sensor responses, meaningful progress has been made. One example is the use of colorimetric sensor arrays in which each spot is tuned to respond differently to various amines through cross-reactive dyes. The resulting response pattern can then be analyzed to infer which amines are most prevalent [66]. Another approach uses mass-sensitive sensors like quartz crystal microbalances (QCMs) with nanomaterial coatings that have affinity for a target gas; the QCM’s resonance frequency shifts proportional to mass of gas absorbed. A nanostructured coating can act as a sponge for a specific gas, achieving a degree of selectivity.

From a logistics viewpoint, gas nanosensors are attractive because they are usually non-destrerefore can often avoid direct contact with the food matrix as well as any sample pretreatment. This is a substantial practical advantage over many off-line analytical methods. However, their deployment trade-offs differ by platform. Passive colorimetric gas labels can be low-cost and simple, but often provide only semi-quantitative outputs. Chemiresistive or NFC/RFID-enabled gas sensors offer faster and more quantitative tracking, yet they rely more heavily on encapsulation design, calibration against humidity and interfering volatiles, and access to an electronic reader. In practice, the key operational question is whether the gas signal remains sufficiently selective and stable to support shelf-life decisions rather than merely indicating the presence of volatiles. For this reason, future deployment should prioritize gas-permeable but liquid-blocking package integration, cross-sensitivity control, and calibration against accepted spoilage benchmarks such as microbial count, TVB-N, or validated freshness classes.

### Biosensors for pathogens and spoilage organisms

While chemical indicators like gases and pH are indirect measures of spoilage, biosensors aim to detect the presence or activity of specific microorganisms or biochemical agents of spoilage. In food logistics, this could mean a sensor that detects *Salmonella bacteria* on poultry, or one that measures the activity of an enzyme. Nanomaterials contribute to biosensors in multiple ways: as immobilization platforms for biorecognition elements, as signal amplifiers, and as part of transducers.

A classic biosensor format is an electrochemical immunosensor, where antibodies specific to a bacterial antigen are attached to a nanomaterial-coated electrode. Magnetic nanoparticles are often used upstream to capture the target bacteria from the sample, then brought to the electrode by a magnet, effectively pre-concentrating the analyte. The electrode itself might be modified with a nanomaterial like gold nanoparticles or graphene to increase its surface area and electron transfer efficiency. Once bacteria bind through the antibody capture layer, the sensor can generate a measurable signal, for example through an enzyme-amplified readout or a detectable change in electrical impedance. Ivanov et al. [9] reported a reusable immunosensor for the mycotoxin aflatoxin B1, where a hybrid gold–magnetic nanoparticle electrode allowed them to not only detect ultra-low levels (LOD ~0.07 ng/mL) but also regenerate the sensor for ~15 uses by magnetically removing the bound toxins [9]. While that example is a safety-related sensor, the principle extends to spoilage microbes: one could detect a sudden bloom of lactic acid bacteria in a juice or *Listeria* on packaged salad if the sensor is designed for those targets.

Another powerful technique is electrochemical impedance spectroscopy (EIS), which measures the impedance of a sensor electrode over a range of frequencies. A label-free impedance biosensor can detect bacteria attaching to an electrode surface by the change in the electrical double layer and charge transfer resistance. If the electrode is a nanomaterial, even a small number of bacterial cells can noticeably alter the impedance [67, 68]. Combining EIS with nanostructured electrodes and specific binding molecules has yielded sensors that detect, for example, *E. coli* in meat broth within minutes at low 10^2^–10^3^ CFU/mL levels [69]. Such sensors could be integrated at points in the supply chain where contamination risk is highest.

For enzyme-based spoilage sensors, nanomaterials often serve as transducers or substrates. A simple example: to detect increasing total volatile basic nitrogen (TVB-N) in fish, one can use an enzyme that reacts with those amines and produces an electroactive product. By coating that enzyme on a nano-porous electrode, the current produced correlates to amine concentration [70]. Alternatively, enzymes naturally present in foods can be coupled with nanostructured electrodes to directly monitor those metabolic processes as the fish spoils. Some researchers have developed printed paper-based biosensors that incorporate nanomaterials. For instance, a paper strip with immobilized acetylcholinesterase enzyme and copper nanoclusters that glow under UV only when the enzyme is active; if microbes produce cholinesterase inhibitors as they grow, the glow diminishes, signaling spoilage. This is a bit niche, but it shows how combining biochemical reactions with nanomaterial signals can yield a functional sensor.

One burgeoning area is DNA biosensors for spoilage organisms, where nanomaterials are used in nucleic acid amplification and detection. Portable PCR devices or isothermal amplification chips can be placed in processing facilities to test for specific spoilage bacteria. Gold nanoparticles functionalized with DNA probes, for example, have been used to visually detect bacterial DNA. These are not yet inline sensors, but they drastically cut the time of microbial testing from days to hours or less, which can inform logistics decisions.

It is worth noting that biosensors generally offer the highest analytical specificity among the sensor classes discussed here, because they rely on selective ban indirect chemical proxies alone. This makes them attractive when the analytical objective is confirmation of pathogen presence, toxin formation, or a specific spoilage pathway. However, that specificity is obtained at the cost of greater practical complexity. Compared with optical labels and many gas sensors, biosensors more often depend on controlled interfaces, calibrated electrochemical measurement, or supporting instrumentation, and in some formats may still require partial sample extraction or localized contact with condensate or food exudate. Their material design must therefore balance signal amplification with bioreceptor stability, food-contact safety, and storage robustness. For logistics use, biosensors are best positioned as confirmatory or high-value monitoring tools unless future formats can demonstrate stable shelf-life prediction, low-maintenance operation, and cost-compatible deployment at package scale.

One illustrative example of a smart logistic application is a microneedle sensor array recently proposed for detecting foodborne pathogens inside bulk packages: tiny needle-like nanoparticle-coated sensors can be inserted into a block of cheese or meat and left there; they change color or electrical signal if bacterial toxins increase [71]. This is akin to a biosensor patch monitoring the food internally. While still experimental, it shows the creative possibilities at the intersection of nanomaterials and biosensing in food.

**Fig. 3:**
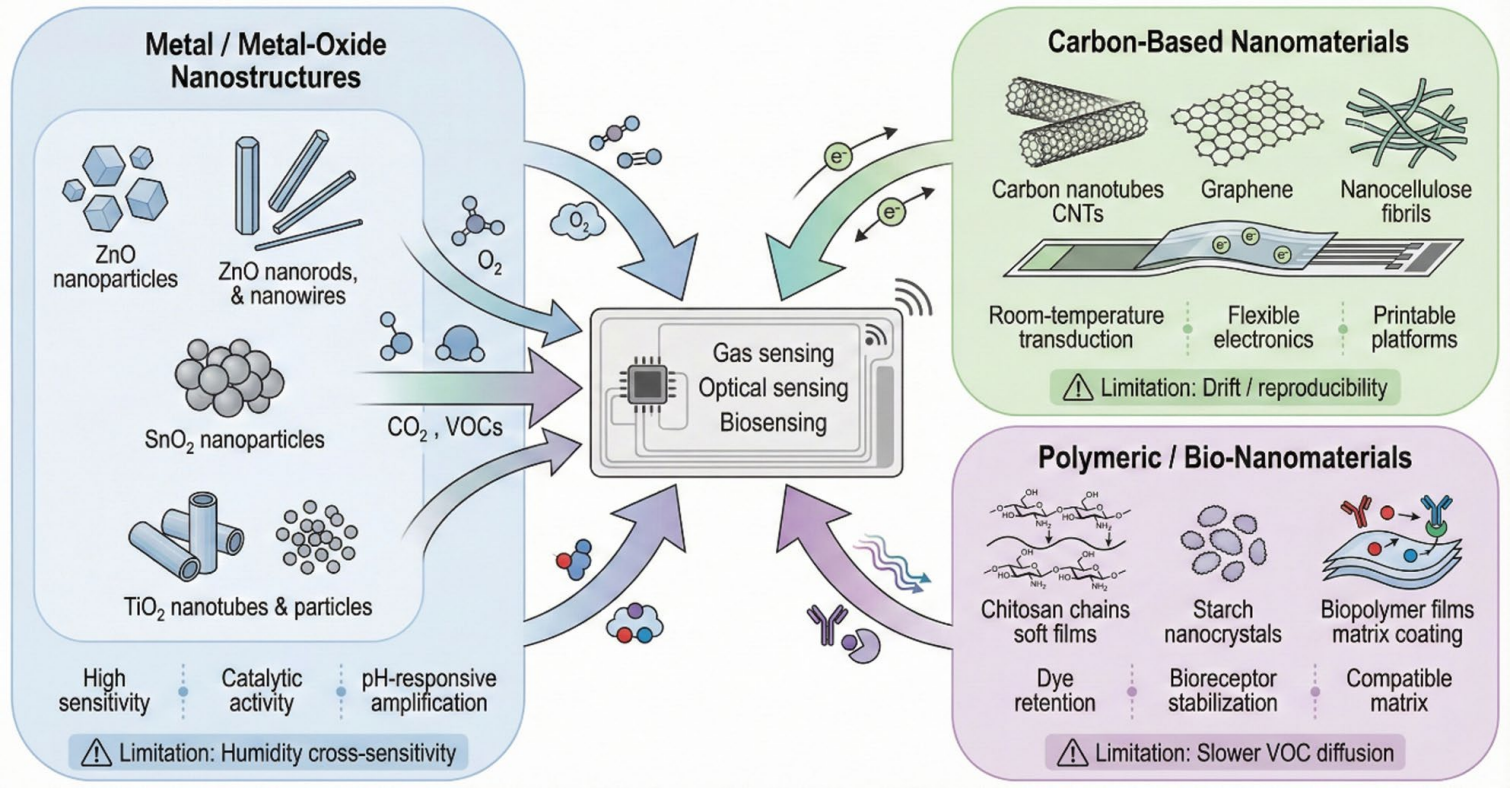
Primary categories of nanomaterials in intelligent packaging sensors

In practice, incorporating biosensors into packaging is challenging due to the need for keeping the bioreceptor alive/functional. Some active packaging concepts include oxygen sensors to ensure modified-atmosphere packs haven’t been compromised. For example, an O₂ sensor spot can alert if a package’s integrity fails and oxygen leaks in, which would accelerate spoilage [14]. Similarly, CO₂ sensors are used for controlled atmosphere fruit shipments to ensure CO₂ stays in range. These sensors benefit from nanomaterials for stable and reversible signaling. Figure 3 synthesizes the material “toolbox” that underpins the sensor concepts in Sect.  "Optical sensors: colorimetric and fluorescent indicators"–"Biosensors for pathogens and spoilage organisms". Metal/metal-oxide nanostructures (e.g., ZnO, SnO₂, TiO₂) dominate chemiresistive gas sensing and also act as catalytic or pH-active components that sharpen colorimetric transitions, but their surface reactivity introduces cross-sensitivity to humidity and interfering volatiles. Carbon-based nanomaterials (CNTs, graphene, nanocellulose) are the key enablers for room-temperature signal transduction and printed, flexible electronics; however, their selectivity is largely “engineered” via surface functionalization or polymer over-layers, which shifts the design problem from raw sensitivity to reproducible fabrication and drift control. Polymeric and bio-nanomaterials (chitosan, starch nanocrystals, biopolymer films) provide the mechanically and chemically compatible matrices that prevent dye leaching and stabilize bioreceptors, yet they can slow mass transport of VOCs and therefore must balance barrier performance with response time. Taken together, these differences indicate that sensor selection for smart logistics should not be judged solely by analytical sensitivity. In practice, deployment also depends on substrate sustainability, the degree of direct or indirect food contact, sample-handling requirements, reliance on external readout hardware, the strength of correlation between sensor output and actionable shelf-life decisions, and overall implementation cost. To provide a clearer cross-platform comparison, Table 2 summarizes the major practical characteristics, advantages, and constraints of representative nanomaterial-enabled optical sensors, gas sensors, and biosensors discussed in Sect.  "Optical sensors: colorimetric and fluorescent indicators"–"Biosensors for pathogens and spoilage organisms".

**Table 2:** Practical comparison of nanomaterial-based optical sensors, gas sensoristics of perishable goods

Sensor class	Representative materials	Sustainability / biodegradability	Food contact mode	Sample pretreatment	Instrument reliance	Shelf-life linkage	Cost / scale outlook	Main practical limitation
Optical colorimetric sensors	Anthocyanin-biopolymer films, PDA/alginate beads, ZnO-assisted dye systems	Often favorable when based on alginate, starch, chitosan, cellulose, or natural pigments; weaker when metal nanoparticles dominate	Can be direct-contact, inner-label, or headspace label	Usually none	Low for visual labels; moderate if smartphone quantification is used	Usually indirect but practical when calibrated to pH, amines, TVB-N, or microbial trends	Low to moderate; scalable as labels/films	Humidity sensitivity, dye migration/leaching, semi-quantitative output
Optical fluorescent sensors	QDs, carbon dots, fluorescent probes in polymer matrices	Variable; carbon-dot systems may be more benign than heavy-metal QDs, but sustainability depends on matrix	Usually headspace label or isolated sensing spot rather than unrestricted direct contact	Usually none	Moderate to high because excitation and image capture are needed	Can be stronger than simple colorimetry when ratiometric or app-based, but still needs calibration	Moderate	Reader dependence, light-source requirement, regulatory concern for some nanomaterials
Gas sensors / e-noses	ZnO, SnO₂, WO₃, CNTs, graphene, printed conductive films	Moderate; can use paper/cellulose supports, but sensing phase often remains inorganic or synthetic	Typically headspace-only; often separated from direct food contact by membrane/barrier	None for in-package sensing	Moderate to high for chemiresistive, RFID, NFC, or e-nose systems	Strong for volatile-driven spoilage if calibrated to product-specific freshness indices	Moderate; printable and batteryless formats are promising	Cross-sensitivity to humidity/interferents, calibration drift, reader dependence
Biosensors	AuNP/graphene electrodes, magnetic nanoparticles, enzyme or antibody interfaces, EIS platforms	Usually less favorable than simple labels because functional biorecognition layers and electronics increase material complexity	Often localized contact with exudate/condensate or test-strip/sample interface; less often simple passive in-package deployment	Low to moderate, depending on format	Moderate to high	Strongest for specific hazard confirmation, but often weaker for simple package-level continuous shelf-life display	Moderate to high	Bioreceptor fragility, calibration burden, shorter operational stability, higher system complexity

### Integration into smart packaging and IoT systems

While individual sensor technologies are impressive in the lab, their value multiplies when integrated into the packaging and logistics infrastructure. Smart logistics requires that sensors are not only present, but that their data is collectable, interpretable, and actionable across the supply chain. In this section, we explore how nanomaterial-based sensors are being incorporated into actual packaging materials or tag devices, and how the data flows from the package to stakeholders. Integration touches on form factor, networking, and analytics.

Intelligent Packaging Formats: One approach is to embed sensors directly into packaging films, cartons, or containers so that the *package itself* becomes the sensing interface. In perishable logistics, the most actionable headspace targets are spoilage- and ripening-related volatiles, particularly ammonia (NH₃) and trimethylamine (TMA) for protein-rich foods (meat/fish), CO₂ as a respiration/fermentation proxy, and ethylene for climacteric fruit ripening. A representative integration route is the use of printed, paper/cellulose-based chemiresistive gas sensors, in which adsorption of water-soluble/basic gases onto hygroscopic cellulose and conductive carbon networks modulates ionic/electronic pathways and produces a measurable resistance change at room temperature [72, 73]. In the literature, near-zero-cost paper-based electrical gas sensors have demonstrated sub-ppm sensitivity for NH₃ (reported limits down to < 0.2 ppm under relevant conditions) and have been positioned specifically for in-package freshness monitoring of meat/fish headspace [72, 74].

Commercial active-packaging pilots are now exploring this same integration logic at carton scale. For example, Solidus and BlakBear have reported a fiber-based carton concept in which a compact freshness-sensing element is embedded into the packaging format and the resulting signals are communicated through RFID and/or Bluetooth-enabled infrastructure for dashboard-level monitoring [75]. While such pilots are valuable for demonstrating manufacturability and logistics workflow fit, they also highlight key constraints that must be managed in deployment, including humidity-driven cross-sensitivity (especially for amine sensing), calibration transferability across products/headspace volumes, and the need for transparent validation against accepted spoilage benchmarks (e.g., TVB-N and microbiological counts) [76, 77].

A more straightforward integration strategy is to use a sensor label or tag (sticker/insert) that contains the sensing element plus a wireless interface. In paper-based RFID/NFC approaches, the label typically combines a printed conductive pattern (e.g., carbon-based electrodes or CNT/graphene-containing inks) with a passive RFID/NFC circuit so that scanning both powers the tag and retrieves a gas- or environment-dependent electrical parameter. The main strengths of this route are low unit cost, compatibility with roll-to-roll printing, and batteryless operation, which is essential for item-level scaling [78]. The drawbacks are that readout remains constrained by reader proximity/infrastructure, and the sensor response can drift due to aging/fouling or varying humidity, requiring calibration strategies and, ideally, multi-parameter sensing or model-based compensation when translating signals into remaining shelf-life decisions [79, 80].

For wireless integration, there are primarily two modes: RFID/NFC passive tags and active/battery-powered transmitters. Passive RFID tags, powered by the scanning device’s radio-frequency field, are attractive due to not needing batteries. Many researchers have demonstrated passive RFID sensors by modifying a standard RFID tag’s antenna or circuitry with a sensing material so that its radiofrequency response shifts in response to environmental changes. One example is an RFID tag whose antenna is partially printed with a carbon nanotube ink; when it absorbs amines and its resistance changes, the tuning of the antenna shifts, which the RFID reader can detect as a change in the backscattered signal [81]. This method turns a simple RFID label into a wireless gas sensor. In 2019, MIT researchers unveiled a passive RFID sensor for milk spoilage that could be read with a smartphone. It incorporated a miniature chemically responsive capacitor. As milk spoiled and pH decreased due to lactic acid bacteria activity, the dielectric properties shifted, causing a change in the RFID tag’s resonant frequency that could be correlated with the degree of spoilage [82, 83]. These examples highlight that nanomaterials can be integrated with RFID to create wireless, battery-free sensor tags.

Active devices, which have a small battery or draw energy from a larger source, can provide continuous monitoring and longer-range communication. For instance, a refrigerated truck might have an on-board system that continuously polls all sensor tags via BLE and uploads the data via cellular network to the cloud. Active tags can also incorporate displays or indicators for human-readable output; for example, Timestrip’s electronic time-temperature indicators have an LCD that shows how much time at temperature has elapsed. If nanotechnology-based sensors are integrated into packaging, one could envision an active tag on the outside of a box that directly displays “Fresh,” “Spoiling,” or a remaining-days countdown based on internal sensor readings, thereby translating raw signals into actionable information at a glance.

The IOT aspect means that sensor data is not confined to individual packages. Instead, it can be aggregated across shipments and analyzed centrally for monitoring, decision-making, and traceability. Cloud platforms are being developed to handle streams of data from thousands of sensor-equipped packages. The FreshIoT concept, for example, involves real-time dashboards that show color-coded statuses of all shipments, analytics that predict shelf life remaining for each batch, and automatic alerts if any consignment shows unusual readings [84, 85]. Advanced analytics could even combine sensor data with external data to perform shelf-life modeling on the fly. For instance, if a pallet’s sensor indicates mildly elevated CO₂ and the system knows the pallet got a bit warm for 2 h yesterday, it might calculate that this pallet’s apples will ripen 1.5 days sooner than expected, prompting the system to suggest rerouting those apples to a closer market or discounting them for faster sale. This is the “analytical control” loop in action: sensors feed data to algorithms, which feed decisions to humans or automated actuators.

A concrete case study is the Solidus–BlakBear trial announced in 2025: fiber-based boxes with embedded freshness sensors were used to pack meat products. As those boxes moved through the supply chain, their sensors continuously monitored spoilage gases and temperature, and the data was sent to a web dashboard in real time. This allowed the producer and retailer to see exactly which batches were nearing end-of-life. According to the companies, even a single extra day of shelf life realized by this technology could significantly reduce waste in retail. Moreover, issues such as a broken cold chain can be detected immediately. If one box shows an unusually high spoilage signal relative to other units from the same lot, it may indicate temperature abuse or contamination, enabling targeted quality control such as inspection or diversion of that unit rather than discarding the entire lot. This kind of granular insight is unprecedented in traditional logistics.

Another aspect of integration is consumer interaction. With smart packaging sensors, consumers could potentially get information about freshness at point-of-sale or at home. For example, by scanning a QR code or tapping an NFC tag on a package with their smartphone, a consumer might see “Packaged on Jan 1, Estimated Fresh Until Jan 8 if stored at 4°C” updated in real time by the sensor [36, 86]. BlakBear’s web app in trials would even send smartphone notifications like “Your chicken is 2 days from spoilage” based on the sensor data. This could transform how food is managed at home. Rather than relying solely on printed dates or disregarding them altogether, consumers could use objective freshness readings to guide safer and less wasteful decisions.

To close this section, it’s clear that integrating nanomaterial sensors into packaging and logistics systems is the lynchpin that makes all the brilliant sensing technologies actually usable in the real world. The major challenges in integration are cost, regulatory approval, and data management. Costs are coming down with advances in printing and the use of cheap substrate materials like paper. Regulatory concerns focus on sensor materials in contact with food. Data management and interpretation require significant backend investment and agreement on standards. There is also the issue of interoperability: ideally, sensors from different suppliers should feed into common platforms so that a retailer doesn’t need separate systems for each sensor type.

Nonetheless, pilot implementations are proving feasibility. As of 2025, companies like Timestrip, Insignia, BlakBear, and others have either commercial products or advanced prototypes of smart freshness sensors in logistics. Some hurdles remain before ubiquitous deployment (discussed next), but the integration of nanotech sensors into IoT-based smart logistics has moved from concept to reality in select supply chains, heralding a new era where every perishable item can effectively “tell” its handler how fresh it is [87, 88].

## Challenges, controversies, and future perspectives

Despite the promise of nanomaterial-based sensing in perishable logistics, significant challenges and points of contention remain. In this section, we critically evaluate the main pros and cons of these technologies, the arguments put forth by proponents and skeptics, and the current focus of debate in the field. The discussion spans technical hurdles, safety and regulatory issues, economic feasibility, and acceptance by industry and consumers. We also explore future directions that address these challenges, mapping out what advancements or frameworks are needed to fully realize smart perishable logistics.

### Sensor performance and reliability

One contested issue is the real-world performance of nano-sensors outside the lab. Proponents highlight laboratory results showing detection of spoilage markers at exceedingly low levels and rapid response times [89, 90]. However, skeptics note that translating this performance to a dynamic, noisy supply chain environment is non-trivial [91, 92]. For example, a sensor might be very sensitive to ammonia, but in a refrigerated truck the background conditions could interfere. As Dr. Ken Suslick argues, some simple sensors lack specificity and can be confounded by unrelated volatiles or changes in humidity [93]. Indeed, a cheap paper sensor might respond to any base gas, not just fish amines, and high humidity can swamp a paper-based reading. Those skeptical of current technology often point to the need for calibration and error rates: how often do these sensors trigger false alarms or miss spoilage? In a supply chain, false positives could cause unnecessary loss of food, while false negatives are a safety risk.

From the limited field trials reported, performance is mixed. The pH indicator films and TTI labels have been in use for years in some sectors, and while they generally work, they sometimes indicate spoilage when quality is still acceptable or vice versa under certain conditions [94, 95]. Newer nano-sensors claim better correlation to actual microbial counts [96]. For instance, BlakBear reports ~ 90% correlation between their sensor’s readings and standard microbiological spoilage tests in meat trials. That is encouraging, but independent validation data are sparse in the public domain. Thus, one controversy is how much one can trust these sensors as a single point of truth. Food companies and regulators will need convincing through rigorous validation that sensor readings truly represent product safety/quality status. In this vein, research is focusing on developing multiplexed sensors to improve reliability. As one article put it, “food spoilage is complex; a multi-parameter sensing approach might be necessary for confidence” [97].

The stability and shelf life of sensors themselves is another concern. A freshness sensor must often sit in/on a package for days or weeks until it’s read. Will its nanomaterial-based components remain stable over that time, especially under stress? Some colorimetric dyes may bleed or fade; some nanomaterial coatings might foul. For instance, metal oxide gas sensors can gradually lose sensitivity if they adsorb contaminants or if their surface chemistry changes over time. Polymer-based sensors might drift. Manufacturers address this by shelf-life testing of the sensor labels: BlakBear’s labels, for example, have been tested to have at least a year of stability before use [98]. But any need for special handling adds complexity.

### Safety and regulatory hurdles

Perhaps the most intense area of debate is the safety of nanomaterials in food applications. By their nature, nanoparticles have high reactivity and can cross biological barriers. Critics worry about toxicological risks if these materials were to migrate from packaging into the food or be inhaled/ingested by consumers. AgNPs, for instance, are widely known for antimicrobial properties and are used in some food storage products, but studies have shown that AgNPs can cause oxidative stress, inflammation, and even DNA damage in mammalian cells under certain conditions [99, 100]. Although regulatory agencies like the US FDA and EFSA have listed some nanomaterials as Generally Recognized As Safe (e.g., nano TiO₂ in certain uses), they caution that safety depends on concentration, particle size, and context [101, 102]. For example, ZnO and TiO₂ nanoparticles are approved as food additives in limited amounts (TiO₂ was used as a whitener in candies, though recently EFSA withdrew TiO₂’s food additive approval in the EU due to uncertainty about chronic effects). At higher doses or as free particles, they can be cytotoxic [103, 104].

In practice, safety risk assessment for nano-enabled sensing/packaging materials follows a hazard–exposure framework in which migration/release is the pivotal exposure determinant, and is therefore evaluated under standardized (often worst-case) food-simulant conditions as recommended in EFSA’s nano-specific risk-assessment guidance. Representative migration studies on nanosilver-enabled food-contact plastics report total silver release typically in the ng/cm² range, with higher values commonly observed in acidic simulants (e.g., 3% acetic acid), underscoring why simulant choice, contact time/temperature, and whether silver is present as ions vs. particulate forms must be resolved analytically when estimating consumer exposure. In parallel, regulatory-scientific reviews highlight that risk conclusions can change when mechanistic endpoints (e.g., genotoxicity potential) remain uncertain, as illustrated by EFSA’s conclusion that TiO₂ (E171) could no longer be considered safe as a food additive because a genotoxicity concern could not be ruled out. Accordingly, for the main nanomaterials discussed in this review (Ag-based components, metal oxides, and carbon nanomaterials), the most defensible pathway toward deployment is to pair “safe-by-design” immobilization/encapsulation with quantitative migration data and nano-relevant toxicology sufficient to support exposure-based margins of safety [105–107].

One argument from the pro-nanotech side is that, in intelligent packaging, nanomaterials can be immobilized or encapsulated to prevent migration. Techniques like incorporating NPs in a polymer matrix or coating them with inert layers (silica, polymer) can reduce leaching [108, 109]. For instance, one can embed AgNPs in a bio-polymer film such that they do not detach but still exert antimicrobial action; similarly, a colorimetric sensor spot can be behind a permeable barrier layer that allows gases but not nanoparticles to pass. Scholars have suggested a “safe-by-design” approach: use larger nano-sized particles and favor inorganic fillers that are generally inert. Moreover, many intelligent packaging designs position the sensor on the inner side of the package and attach it to the interior surface, so it is exposed to headspace gases without directly mixing with the food. Regulators therefore recommend migration testing under worst-case conditions to confirm that any leached substances remain below established safety thresholds.

Regulatory frameworks for nano-packaging are still evolving. There is a “fragmented and underdeveloped” environment globally [110]. The EU requires that any use of engineered nanomaterials in food contact materials be explicitly approved and labeled if they migrate, but enforcement is tricky because how do you define a nanomaterial legally? The definition by size (1–100 nm) is one approach, but functionality might matter more. The US FDA evaluates nanoscale additives on a case-by-case basis but has no blanket nanotech packaging regulation yet [111]. This regulatory uncertainty has been cited by industry as a barrier, since companies may be reluctant to invest in nanosensor packaging that could conflict with future requirements or trigger consumer backlash. Public perception compounds the issue because “nano” in a food context can raise suspicion. Survey findings are mixed: while many consumers appreciate the promise of fresher and safer products, they also report concerns about potential unknown health effects associated with nanocoatings and nanomaterials used in packaging [112]. Indeed, the term “nanoparticle” could become a marketing red flag unless carefully managed.

To address safety, scientists argue for rigorous risk assessment and communication. Migration studies in simulants are crucial. If results show negligible migration, that should be communicated to regulators and the public. In addition, there is growing emphasis on using food-grade nanomaterials where feasible. For example, naturally derived options such as nanocellulose or nanoclays, which have generally shown low toxicity in many studies, are often favored over more exotic materials such as metal nanoparticles or quantum dots. The idea of biodegradable and edible sensors is even being floated: imagine a tiny tag made of edible polymers and safe colorants that could even be consumed with the food. These would circumvent some regulatory hurdles if proven safe.

Another controversy is whether the introduction of active nanomaterials in packaging might inadvertently affect the food’s properties. For instance, a very potent antimicrobial packaging might overly sterilize a food product, potentially selecting for more resistant strains or altering the natural maturation of foods like cheese or dry-aged beef that rely on microbial or enzymatic action for flavor. This is a nuanced issue: the goal is to prevent spoilage and pathogen growth, but some level of microbial activity is normal. Active packaging must be calibrated so it doesn’t, say, release so much antimicrobial that it could pose a hazard or change the food’s taste. Controlled release systems for antimicrobials, often using nanocarriers, are being researched to ensure only tiny, effective amounts are released over time rather than a big burst. This connects directly to nanomaterials because many antimicrobial packaging systems rely on nano-encapsulated essential oils or nanosilver. In these designs, controlling the release profile is essential to balance antimicrobial efficacy with safety considerations.

### Economic and scale challenges

On the economic front, the key question is cost-benefit ratio. Integrating sensors increases packaging and logistics costs, and even an added few cents per item can be material in the food industry given its typically slim margins. Are the benefits enough to justify these costs? Proponents argue yes: food waste itself is a massive economic loss (nearly 1 trillion globally each year), so even a small reduction in waste could offset sensor costs. One case study estimated that adding a $0.05 sensor to a $10 piece of meat could extend shelf life by one day and prevent the product from being discarded, effectively preserving the full $10 value and delivering a very high return on investment. However, realizing meaningful system-wide waste reduction depends on broad adoption across the supply chain.

Initial adopters might bear higher costs until scale is achieved. Manufacturing nanomaterial sensors at scale is challenging but progress is being made via roll-to-roll printing and ink formulations. Still, skeptics note that many lab demonstrations use lab-made nanomaterials. Achieving commodity pricing for these materials is needed. For example, high-purity single-wall carbon nanotubes are expensive, but if used in sensors maybe one can use lower-grade CNTs or only a tiny amount per sensor. Scalability of nano-synthesis is a known issue: making a batch of lab graphene is one thing, making tons for packaging is another. However, certain nanomaterials like nanoclays or nano-cellulose are already produced in bulk for other industries at low cost. These could be leveraged in packaging as both functional and structural additives.

Another economic consideration is the cost of data logistics. Building an Internet of Things system requires more than the sensor itself, including infrastructure such as readers, connectivity, cloud storage, and analytics software. Who bears that cost, and who reaps the reward? If the benefit primarily is reducing waste at retail, one could argue retailers should invest in it. But if it helps manufacturers optimize their distribution and reduce returns, they benefit too. There may need to be collaborative efforts or service models. This is new territory for the food supply chain, which is traditionally low-tech. The introduction of high-tech systems raises the issue of technical expertise: do grocery store staff or truck drivers need training to use these sensors or interpret data? Ideally, the system automates most decisions, but during a transition period there could be confusion or misuse.

One vocal point of debate is “Is it cheaper to just improve cold chain management than to add sensors?” Some argue that if we simply invested more in reliable refrigeration and handling, much spoilage could be prevented without fancy sensors. Indeed, basic issues like intermittent power, poor insulation, or delays in transport cause a lot of waste in developing countries. However, even an optimized cold chain cannot override biological reality. Food will still spoil over time, and quality can vary substantially across products and lots. Plus, sensors can complement good refrigeration by verifying it. Nonetheless, some supply chain managers question spending on sensors when fundamental infrastructure in some regions is lacking. A balanced view is that both are needed: infrastructure improvements to prevent losses, and sensors to monitor and fine-tune processes as well as catch the inevitable problems that still occur. An analogy often cited is the automobile: better engines and brakes are crucial, but we still put sensors and gauges on the dashboard to monitor performance and catch issues in real time.

### Consumer and industry acceptance

For any innovation in the food sector, consumer perception matters enormously. Intelligent packaging presents an interesting case: if it works well, consumers might never see it. But there’s also a push for consumer-visible freshness indicators to replace or augment date labels. Behavioral studies indicate that many consumers are confused by existing date labels, such as the distinction between “best by” and “use by”. A clearer freshness indicator could therefore help, but only if consumers understand how to interpret it. A green-to-red freshness label seems intuitive (green = fresh, red = bad), yet intermediate colors might be ambiguous and cause either too early disposal or too late. Public education and standardization would be needed. For example, regulators might need to define what a given sensor color means in terms of safety margin.

Privacy is an unlikely issue here, but there is some concern on the industry side about data transparency: if every item is sensor-tracked, a retailer might worry that data could be used against them. On the flip side, it could protect them by proving due diligence. Either way, agreements on data sharing and liability will have to evolve. If an AI system says “sell that product today or throw it” and the retailer follows it, who is liable if something goes wrong? Likely the same as current practice, but if they followed a system’s advice, one might argue the system designer had a role. As these systems become more autonomous, such questions will need addressing.

From an industry standpoint, a major hurdle is standardization. If different companies use different freshness metrics or sensor types, it will be hard to have a consistent system. Efforts are underway to incorporate sensor data into supply chain standards. For instance, encoding a “freshness score” into a barcode or RFID data field that can be universally understood. There is also talk of combining sensor data with blockchain traceability, so each product’s storage history and current condition are logged immutably, enhancing transparency for safety regulators and consumers.

Despite challenges, the momentum toward smarter packaging is driven by global imperatives: reducing food waste, improving food safety, and optimizing supply chains for a growing population. The controversies surrounding cost, safety, and trust mirror those encountered with earlier food technology innovations, such as irradiation and genetically modified organisms, although typically on a smaller scale. The difference here is that intelligent packaging doesn’t alter the food itself, just monitors it, so it may face less philosophical resistance and more practical skepticism. Surveys have shown that when framed as a way to ensure safety and freshness, consumers are broadly positive, but if framed as “nano particles in your packaging,” they react negatively [113]. This highlights the need for careful communication: emphasize benefits and the fact that materials are contained and safe, perhaps avoid the term “nanoparticle” in consumer-facing contexts. Importantly, acceptance is also shaped by the *material and system design choices* used to realize real-time nanosensing in practice. Recent work has moved beyond stand-alone lab prototypes toward deployable formats such as disposable, package-integrated wireless tags and flexible substrates. For example, NFC-enabled wireless gas-sensing labels have been demonstrated for continuous spoilage monitoring in realistic packaging workflows, enabling smartphone-based access without additional readers or sample handling [114]. In parallel, flexible chemiresistive sensors leveraging nanocomposites that combine carbon nanostructures with porous frameworks have enabled room-temperature ethylene monitoring for fruit freshness; a representative example achieved ppb-level ethylene detection and real-time tracking of kiwifruit ripening using a SWCNT/PdNP/Cu-MOF-74 composite on a flexible substrate [115]. Real-time freshness indicators based on nanostructured colorimetric assemblies are also advancing, such as polydiacetylene-based systems that respond to biogenic amines from meat spoilage [116]. Finally, emerging 2D materials (e.g., MXene-based paper/flexible sensor architectures) are being actively developed for low-temperature/room-temperature ammonia sensing, which is directly relevant to amine-rich spoilage headspaces [117]. These examples strengthen the linkage between “real-time nanosensors” and adoption drivers: low-power readout (NFC), manufacturable flexible/paper substrates, and target-relevant gases (amines/ethylene) reduce operational friction for industry and help keep the sensing layer physically isolated from food contact, mitigating consumer concerns.

**Fig. 4:**
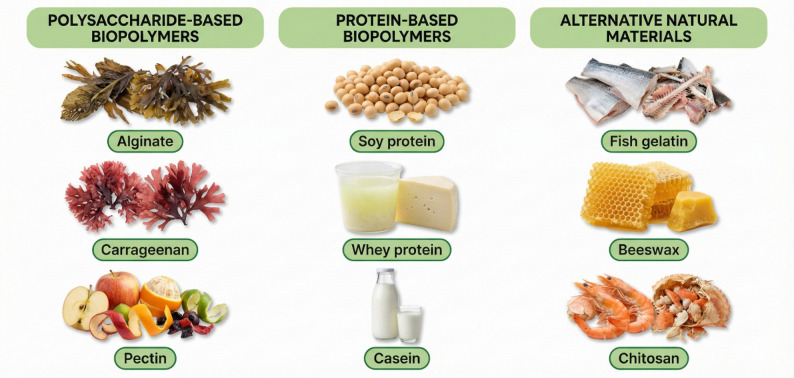
Biopolymer and natural sources for sustainable nanomaterials in smart packaging

Looking ahead, several key future directions can be identified to address current limitations:

- Improved Selectivity and Multi-Sensor Fusion: Research is focusing on hybrid sensors to minimize false readings and capture a fuller picture of food quality. Advances in material science, like MIPs (molecularly imprinted polymers) on nanomaterials for specific analytes, or 2D materials (e.g., MXenes) with high sensitivity to certain gases, will continue to improve sensor specificity.
- Standardized Calibration and Algorithms: There is a need for industry standards on what sensor readings correlate to in terms of remaining shelf life or microbial levels. Future systems will likely employ AI and machine learning trained on big data from many sensor deployments to predict spoilage more accurately. These algorithms can be shared or certified, much like thermometers are calibrated to standards.
- Integrating Activeness with Intelligence: A visionary outlook is packaging that not only senses but also responds. For instance, if a sensor detects rising CO₂ (indicating ripening fruit), the package could actively release a CO₂ absorber or an ethylene scavenger to slow further ripening. Some experiments have involved packaging that, upon detecting a threshold of spoilage, releases a burst of preservative (like an antimicrobial essential oil) to delay the spoilage – essentially giving the food an extension of life as soon as it starts to turn. Nanotechnology could enable such triggered release systems.
- Biodegradable and Sustainable Sensor Materials: To align with sustainability goals, future sensors are expected to incorporate more environmentally friendly nanomaterials, such as biopolymer-based nanofibers, nanocellulose, or graphene derived from biomass and other renewable feedstocks [118]. We may see fully biodegradable sensor tags so that the increased use of sensors doesn’t itself contribute to electronic or plastic waste. Figure 4 in the reference conceptually illustrates “green sources” for nano-enabled packaging, such as starch, chitosan, and gelatin, highlighting the broader push toward renewable and bio-based materials in the next generation of smart packaging. As shown in Fig. 4, biopolymer-derived nanomaterials (e.g., cellulose nanofibers, chitosan, and starch nanocrystals) provide a ‘safe-by-design’ substrate for intelligent packaging, because they are inherently food-contact compatible and can be engineered to be compostable at end-of-life. Critically, these matrices do not only reduce environmental burden; they also influence sensing fidelity in logistics. Their hydrophilicity and porosity govern VOC diffusion and water uptake, which can shift response kinetics and baseline drift, especially under cold-chain humidity excursions. Therefore, future work should couple these sustainable matrices with controlled mass-transport architectures (barrier/porous bilayers) and calibration against reference freshness metrics, ensuring that greener materials improve both regulatory acceptance and measurement robustness rather than trading one for the other.

Regulatory Frameworks and Collaboration: A key future step is establishing clear guidelines for intelligent packaging. Efforts like the EU’s Active and Intelligent Packaging regulation (which allows such packaging given it doesn’t mislead consumers and any released substances are safe) need to be harmonized globally. Industry consortia are likely to collaborate with regulators to establish clear testing and compliance protocols for nanosensor packaging, including standardized migration testing, defined safety factors, and practical labeling requirements. Collaboration between sensor developers and food companies in large-scale pilots will build the safety and efficacy evidence base needed for broad approval.

In conclusion, the intersection of nanotechnology and food logistics, while still facing growing pains, is poised to significantly enhance the sustainability and safety of perishable supply chains. Proponents argue that with continuing innovation and proper safeguards, nano-enabled sensors will drastically cut waste and improve consumer confidence in product quality. Critics urge caution, pointing out that without careful design these sensors could introduce new risks or simply add cost without commensurate benefit if not highly reliable. The current focus of controversy centers on proving that these smart sensors are accurate and cost-effective enough to become industry standard, and ensuring any risks are mitigated. Many in the field recognize that earning trust from regulators, industry stakeholders, and consumers is just as critical as achieving technical performance. This will require transparency in communicating how the sensors work, independent validations, and probably starting with “low-hanging fruit” applications that clearly demonstrate value.

The coming years will likely see intelligent packaging move from niche pilots to more common usage, as the pressure to reduce losses and ensure food safety grows. The path will involve resolving the challenges discussed: refining sensor accuracy, reducing costs through scale and printing tech, addressing safety via safe-by-design nanomaterials, and establishing standards. The potential rewards are compelling: a food system in which individual items are monitored and maintained at optimal quality, spoilage-related surprises are minimized, and the notion of “expired” becomes increasingly outdated because real-time data can indicate whether food remains acceptable. Smart logistics, underpinned by nanomaterial sensors, thus represents a transformative leap toward a more sustainable and efficient future for perishable goods management.

## Conclusion

Perishable-goods logistics cannot rely on temperature logs and static date codes alone, because deterioration is product- and history-dependent and food loss and waste remain substantial at the system level. This review shows that nanomaterial-enabled sensing is most impactful when it converts package headspace chemistry and microbial activity into decision-ready freshness information that can be captured at scale. Across the literature, three complementary technology families dominate: (i) optical indicators (e.g., pH/amine-responsive nanostructured dyes and fluorescence probes) that offer intuitive visual or camera-readable outputs; (ii) gas sensors/e-noses based on metal-oxide and carbon nanostructures that quantify spoilage VOCs (amines, sulfur compounds) and ripening gases (ethylene, CO₂) in real time; and (iii) biosensors that add analytical specificity for pathogens, toxins, or biomolecular spoilage markers. Translation to “smart logistics” depends less on peak analytical sensitivity than on robust calibration under humidity and matrix variability, validated linkage to accepted freshness benchmarks, and deployment-ready readout pathways (e.g., RFID/NFC and low-power wireless tags) that support remaining-shelf-life analytics and operational control. Therefore, near-term progress should prioritize multi-parameter designs with drift compensation, interoperable data standards, and safe-by-design packaging integration supported by quantitative migration/safety evidence, so that sensor outputs can be trusted by regulators, supply-chain operators, and consumers.

## Data Availability

The datasets used and/or analysed during the current study are available from the corresponding author on reasonable request.
